# The Density Functional Theory Account of Interplaying Long-Range Exchange and Dispersion Effects in Supramolecular Assemblies of Aromatic Hydrocarbons with Spin

**DOI:** 10.3390/molecules27010045

**Published:** 2021-12-22

**Authors:** Ana Maria Toader, Maria Cristina Buta, Alice Mischie, Mihai V. Putz, Fanica Cimpoesu

**Affiliations:** 1 Institute of Physical Chemistry, 060021 Bucharest, Romania; atoader@icf.ro (A.M.T.); butamariacristina@gmail.com (M.C.B.); alice_mischie@yahoo.com (A.M.); 2 Laboratory of Computational and Structural Physical-Chemistry for Nanosciences and QSAR, Biology-Chemistry Department, Faculty of Chemistry, Biology, Geography, West University of Timisoara, 300115 Timisoara, Romania

**Keywords:** density functional theory, long-range interactions, organic radicals

## Abstract

Aromatic hydrocarbons with fused benzene rings and regular triangular shapes, called *n*-triangulenes according to the number of rings on one edge, form groundstates with *n*-1 unpaired spins because of topological reasons. Here, we focus on methodological aspects emerging from the density functional theory (DFT) treatments of dimer models of the *n* = 2 triangulene (called also phenalenyl), observing that it poses interesting new problems to the issue of long-range corrections. Namely, the interaction comprises simultaneous spincoupling and van der Waals effects, i.e., a technical conjuncture not considered explicitly in the benchmarks calibrating long-range corrections for the DFT account of supramolecular systems. The academic side of considering dimer models for calculations and related analysis is well mirrored in experimental aspects, and synthetic literature revealed many compounds consisting of stacked phenalenyl cores, with intriguing properties, assignable to their long-range spin coupling. Thus, one may speculate that a thorough study assessing the performance of state-of-the-art DFT procedures has relevance for potential applications in spintronics based on organic compounds.

## 1. Introduction

**Figure 1:**
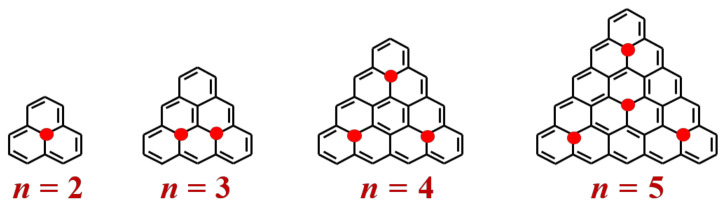
Structures of the first elements of *n*-triangulenes class; *n* is the number of benzene rings at one edge; the open spin sites are represented with red points, taking the resonance structures with the highest possible symmetry.

The field of stable carbon-based radicals [1,2] offers promises for the desiderata of spintronics with organic materials [3,4,5,6] and challenges in the fundamental respects of exotic bonding regimes [7]. The causal roots of special properties (conduction and magnetism) that are making polyaromatic hydrocarbons [8,9] and graphenes [10,11] interesting for materials sciences are common with the bonding factors tied under the heuristic concept of aromaticity [12,13].The delocalization implied by aromatic bonding determines functionalities such as reservoirs and conductors of charge [14,15,16] and spin [3,17].Triangulenes, i.e., condensed aromatic hydrocarbons with regular triangular geometry (spanning D_3h_ point group), are excellent objects for theoretical and experimental studies along the above-mentioned desiderata. By classifying triangulenes by the number *n* of fused benzene rings at one edge, one may point that there are many derivatives of the *n* = 2 core [18,19,20] known also as phenalenyl (see Figure 1 for examples of *n*-triangulenes).The *n* = 3, 4 and 5 are more elusive but firmly characterized by surface techniques [21,22,23]. Larger systems are not yet known, but surface tailoring methods suggest promising future developments.

In previous studies, we scrutinized spincoupling and resonance effects in monomeric [24] and dimeric [25] triangulenes, particularly relying on complete active space (CAS) and valence bond (VB) calculations. Recently [25], we analyzed the full landscape of singlet and triplet states together with related exchange coupling, as a function of separation and mutual rotation of two phenalenyl radicals sharing a common third-order rotation axis. The calculations, made with Complete Active Space (CAS) methods followed by second order corrections (PT2) and, comparatively, with B3LYP density functional, were used as support of an interesting qualitative magneto-structural correlation model. At the same time, a phenomenological VB model was developed.

During our previous investigations, we spotted the interesting potential of triangulene dimers as a new conceptual and practical test of density functional theory (DFT) in non-covalent interactions. Here, we develop the problem in the key of methodological interests for DFT applications in a long-range regime. We present new situations for DFT assessment, considering the fact that phenalenyl supramolecular associations have two interplaying long-range interactions: spin coupling and van der Waals dispersion.

**Figure 2:**
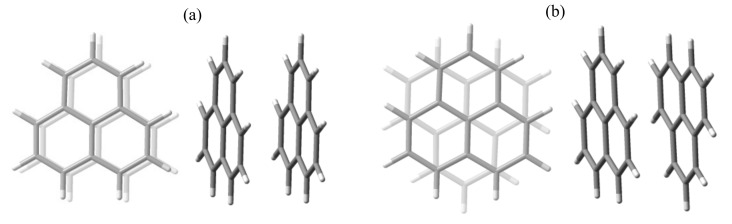
The top and side views of two distinct phenalenyl dimer conformations: (**a**) eclipsed and (**b**) staggered.

In a benchmark on fullerenes incorporating noble gas atoms [26], we showed that long-range correction protocols may be problematic when tested in rather non-standard systems. Thus, considering the phenalenyl dimers shown in Figure 2, we perform a new form of testing for long-range DFT amendments. In the following sections, we focus on staggered conformation because, in experimental instances, it is more frequent than the eclipsed conformation [19]. Another reason for confining ourselves to staggered geometry is because it is expected to render results similar to the eclipsed one, especially in exchange coupling, as computational data from previous results [25] and outlined magneto-structural rationalization supports. Then, we avoid a certain redundancy, as the convened limitation serves the proposed aims well.

One may say that, inside the triangulenes, the problem of topologic spin is mingled with the issue of aromaticity in planar conjugated hydrocarbons [27]. As extension, in supramolecular triangulene complexes, of π–π stacking [28,29] comes together with the problem of long-range exchange coupling [30].

An extreme manifestation is the formation of a σ bond between phenalenyls (loosing mutual planarity of cores) or, most intriguingly, a sort of intermediate stage between σ-linkage and π–π stacking, interpreted as a fluxional σ bond [31,32].In the above-cited studies, computational experiments were correlated with dynamic EPR, NMR and electronic UV-VIS spectra sustaining the idea of a weak σ bond traveling between couples of carbon atoms situated at the periphery of phenalenyl units. In this case, the formation of the σ bond implies the localization of the radical at a certain atom in each monomer, establishing a dimer with tilted aromatic planes.

Taking a series of hypothetical dimers of *n*-triangulenes (with *n* from 2 to 5), Mou and Kertesz [33,34] interpreted the so-called pancake bond order of dimeric associations as similar to orbital schemes in diatomic molecules. An all-covalent phenalenyl dimer having a single inter-unit bond seems to be unstable, since the dimerization ends in establishing two pairs of C–C linkages between units form a planar non-radical molecule (peropyrene), with seven benzene rings (six from former phenalenyls and one created at the junction) [19,27]. However, the single-σ-bonded dimer (biphenalenylidene) was demonstrated as metastable intermediate in the course of peropyrene formation [35].

Several DFT analyses [7,31,32] are considering the biradical nature of the dimeric system correctly. Namely, although the groundstate is a spin singlet, the wave function cannot be taken directly by stating singlet multiplicity in the input directive, because in this manner, even in an unrestricted mode, the calculation evolves toward restricted-type orbitals, with a doubly occupied HOMO (highest occupied molecular orbital) and empty LUMO (lowest unoccupied molecular orbital), resulting from in-phase and out-of-phase combinations of monomer SOMOs (singly occupied molecular orbitals).The physical truth is that the monomers retain localized unpaired one-electron orbitals, a result that can be obtained in the frame of so-called broken-symmetry (BS) treatment [36,37], guiding the calculation by an educated guess.

At the same time, the BS state is not a physical form of the singlet but merely a computation fictional object, enabling information about the exchange of coupling parameter, *J*, in the spirit of the Heisenberg-spin Hamiltonian [38]. Therefore, the true singlet state in phenalenyl dimers cannot be directly obtained from DFT calculation. It can be emulated from a BS treatment, assuming that the singlet is placed at the 2*|J|* energy amount below the unrestricted triplet (*J* being negative). This meaningful detail was overlooked in previously cited studies [7,31,32].

The aimed BS calculations in phenalenyl dimers are operating with DFT in long-range regime. It is wellknown that empirical functionals have incorrect long-range asymptotic behavior [39,40,41,42]. A rational and general correction procedure consists in conventional dichotomization of two-electron terms [42,43,44] into a short-range zone, treated with the density functional, while the long-range counterpart is accounted for in a Hartree–Fock (HF) manner. Such long-range corrections, usually marked by the LC prefix [42], are valid only on pure functional methods. Among the hybrid functionals, one may mention a principle similar to LC-type amendments in the case of Coulomb attenuated method (CAM) [45], associated with the very popular B3LYP [46,47] option. There are many other ideas of long-range alleviation. For example, in the M11 case [48], which is a functional from the Minnesota family, the HF exchange part is preponderant at large *r*, while it is taken in small percentages at shorter distances. The most pragmatic form is the Grimme-alike treatment [49,50,51], by adding empirical van der Waals contributions in the form of inter-atomic inverse-power terms.

The roadmap of the proposed discussion is as follows. In Section 2.1, we observe certain peculiarities of spin density distribution in the phenalenyl radical (i.e., the monomer in the actual context) as a function of different computational settings. In Section 2.2, we discuss the Broken Symmetry (BS) approach, particularized to the considered biradical, in order to propose a new strategy to emulate a corrected low spin (LS) energy formula, as the BS singlet itself is not fully adequate to account for the singlet state of the system. In Section 2.3, we present the formulas proposed to fit key parameters from various calculations in order to organize, in synthetic form, the discussion of the computational results on the extended series of functionals. Section 2.4 includes a picturesque preamble of the main topic, picking three case studies derived from the most popular functional, B3LYP. The results on selected series of functionals, grouped in three classes, are debated in Section 2.5 on the basis of parameters fitting DFT results.

## 2. Results and Discussion

### 2.1. The Phenalenyl Unit as Spin Carrier

First, let us point a quite intriguing fact about the phenalenyl monomer itself. The spin distribution of the phenalenyl unit in unrestricted single determinant mode is different in the Hartree–Fock (HF) vs. Kohn–Sham (KS) comparison. HF is considered in restricted open-shell (ROHF) and unrestricted (UHF) forms, while KS is observed only in the unrestricted mode (UKS). The tests are made with the 6-31+G* basis. ROHF shows only positive spin densities, localized on certain subsets of atoms, due to topological reasons. In principle, the central atom is allowed to carry restricted open-spin density, while quantitatively it is very small. In unrestricted forms, the sites that are not supposed to wear the α spin hold, by spin-polarization mechanisms, the tails of β spin density.

As ROHF results seem incomplete and UHF somewhat flawed (as will be discussed immediately), one may draw the otherwise expected conclusion that DFT (illustrated here by B3LYP functional) performs better than HF. To distinguish from most cases, when DFT proves its virtues in rendering lower total energies, whereas wavefunctions and populations are quite comparable in HF vs. KS computational couples, here, one notes a drastic variation in the basic spin distribution.

**Figure 3:**
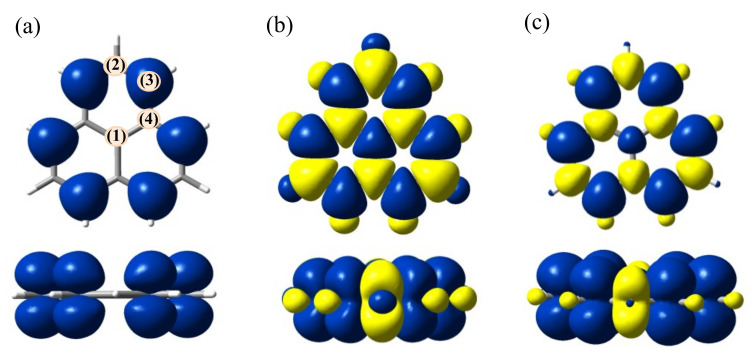
Spin density maps (drawn at 0.001 e/Å^3^isosurfaces) from different single-determinant calculations: (**a**) ROHF (α-only); (**b**) UHF (α-in blue, β-in yellow); (**c**) UKS result, with B3LYP functional. Note that UHF shows a non-physical enhancement of spin polarization, with comparably large α vs. β-zones.

Thus, by UHF, the $\left\langle S^{2}\right\rangle$ expectation value on a phenalenyl molecule is too large, 2.0276, while the DFT produces the reasonable 0.7992 quantity, close enough to the ideal 0.75 value for a spin-doublet state. This situation seems determined by the artificial enhancement of atomic spin components. Figure 3 shows spin polarization maps for HF and DFT (B3LYP) calculations of phenalenyl. One observes that, at the same isosurface threshold, the volumes of alternating α and β densities are larger in the UHF picture (compare Figure 3b with Figure 3c).

The UKS spin map (Figure 3c) is closer to the physical truth, showing correspondingly weighted α vs. β alternation between neighbor atoms, with the predominance of α-type volumes. With atom labeling ((1)–(4)) from panel (a) of Figure 3, the Mulliken spin populations from the UHF calculation (Figure 3b) are as follows: 0.5903, −0.9961, 1.1468 and −1.1232. KS results (panel 3c) are −0.0039, −0.1644, 0.3564 and −0.1917, respectively. One remarks the wild variation of UHF-based positive and negative values, most of them close to the unity, as an absolute magnitude. The abnormal variation of the UHF spin population remains if another analytic scheme is used, such as natural populations in the frame Natural Bond Orbital (NBO) paradigm [52,53], yielding 0.5381, −0.5610, 0.6423 and −0.5316 for the mentioned series. One notices a certain attenuation, but the α vs. β alternation is still too large. NBO populations for UKS, 0.0491, −0.1067, 0.2639 and−0.0910, are roughly comparable with the previously provided Mulliken ones. Thus, one may say that the phenalenyl itself poses interesting questions to subtle aspects of routine calculation procedures.

Although the potential energy curves for phenalenyl dimers were considered in several instances [31,32,33,34], some details remained unobserved, and the following discussion pays the right attention to the implied issues, particularly to certain DFT specificities. In a recent study [25], we considered and rationalized, by magneto-structural orbital models, energy profiles and spin coupling as a function of the general rotation of phenalenyl units around a common C_3_ axis. In the following, in order to debate DFT specific aspects, we are confining ourselves to the study of staggered conformation, with D_3d_ symmetry of the dimer, by varying the inter-planar distance between monomers.

### 2.2. Broken-Symmetry DFT Calculations of the Phenalenyl Dimer

In order to extract information about global long-range exchange coupling, the Broken Symmetry (BS) technique [36,37] will be used, taken in density functional frame, namely as BS-DFT. The method consists in performing unrestricted calculations on different spin multiplicities, trying to impose localized spin polarizations. For a system having two spin carriers, with *S*_1_ and *S*_2_quantum numbers, the aimed configurations are as follows: one is called high spin (HS), with total *S_z_* = *S*_1_ + *S*_2_ projection, and the other is named broken symmetry (BS), with total *S_z_* = *S*_1_ − *S*_2_ projection (assuming *S*_1_ ≥ *S*_2_), which must show localized +*S*_1_ and −*S*_2_ spin projections. For symmetric dimers, the BS solution is not the automatic convergence result, since it lifts the equivalence of the two sites. The BS configuration should be distinguished from a solution with the total spin *S* = |*S*_1_ − *S*_2_| solution, obeying the symmetry, which is conventionally called low spin (LS) state.

The formula [54,55] for extracting the coupling parameter in a system with two spin carriers is:

(1) $$J_{inter}=-\frac{E_{\mathrm{HS}}-E_{\mathrm{BS}}}{\langle {\hat{S}}^{2}\rangle {}_{\mathrm{HS}}-\langle {\hat{S}}^{2}\rangle {}_{\mathrm{BS}}}$$

where the numerator of the fraction uses the computed total energies of BS and HS configurations, while the denominator contains the corresponding expectation values of the spin square operator. All these quantities are picked from the output of the performed unrestricted DFT calculations. We subscripted by inter the exchange coupling symbol in order to suggest its intermolecular nature in the actual discussion.

Concretized for the *S*_1_ = *S*_2_ = 1/2 local spins, a BS regime is described either by the α_1_β_2_ or by β_1_α_2_ single determinants. Convening that the indices 1 and 2 represent orthogonalized orbitals at the two monomer sites, these two configurations form the basis of the formal configuration–interaction Hamiltonian matrix:

(2) $$H_{0}=\left(\begin{array}{cc} E_{0} & J_{inter}\\ J_{inter} & E_{0} \end{array}\right)$$

where *E*_0_ includes all contributions not due to spin coupling (one-electron and two-electron Coulomb terms). The solutions are a singlet and triplet, respectively, and the above-named LS and HS states have the following eigenvalues: *E_LS_* = *E*_0_ + *J_inter_* and *E_HS_* = *E*_0_ − *J_inter_*. At the *S_z_* = 0 projection, the singlet has an (α_1_β_2_ − β_1_α_2_)∙2^−1/2^ eigenvector, while HS consists in an (α_1_β_2_ + β_1_α_2_)∙2^−1/2^ counterpart. The HS set also comprises α_1_α_2_ and β_1_β_2_ configurations, completing the *S_z_* = ±1 componentsof the triplet.

While HS is approachable in DFT as the α_1_α_2_ single determinant, the two-configuration LS wavefunction is outside the DFT frame. In Equation (2), *E*_0_ is actually the idealized BS energy. This simplified scheme tacitly assumes orthogonal orbitals and restricted-type determinants. In this case, since HS energy, *E*_0_ − *J_inter_*, is formally available from the α_1_α_2_ -based calculations, BS-type energy expectation value serves to obtain the coupling by the corresponding difference *J_inte r_* = *E*_0_ − *E_HS_* = *E_B_*_S_ − *E_HS_*. The simplified picture from Equation (2) differs from the conditions described around Equation (1) by the fact that the former frame was conceived for unrestricted determinants and generalized spin values on the sites.

Continuing with the particularized Equation (2) model, the practice shows that both *E*_0_ and *J_inter_* are decaying in exponential patterns with inter-center separation, *R*:

(3) $$E_{0}\to F\cdot e^{-f\cdot R}$$

(4) $$J_{inter}\to -G\cdot e^{-g\cdot R}$$

where $E_{0}$, proceeding to zero at large *R*, undergo tacitly the subtraction of monomer energies. The coupling parameter is imposed as a negative, as it is needed to express a bonded state at the singlet. By introducing Equations (3) and (4) in Equation (2), the eigenvalues are as follows.

(5) $$F\cdot e^{-f\cdot R}\pm \;G\cdot e^{-g\cdot R}$$

One may observe that, at a certain balance of parameters, the above equation turns into a couple of Morse and anti-Morse curves, made explicit in Equations (11) and (12) in the next section. The particular conditions for such a coincidence are as follows.

(6) f=2a

(7) g=a

(8) $$F=D\cdot e^{2aR_{0}}$$

(9) $$G=2D\cdot e^{aR_{0}}$$

There is no reason to propose such constrains, as the Morse curve itself [56,57] has no first-principles justification, except for the practical utility of its particular shape, with a transparent relation between bonding parameters (energy and equilibrium radius) and the visual features of the potential.

The above divagation may serve to persuade the point that a certain part of the observed bonding effect is due to a spin-coupling, for which its parameters are revealed in the course of BS treatment. The overall energy profiles include other contributions that cannot be transparently equated, namely the rather mysterious dispersion effects. However, knowing the part due to spin coupling from the BS analysis, one may dichotomize exchange and dispersion components.

**Figure 4:**
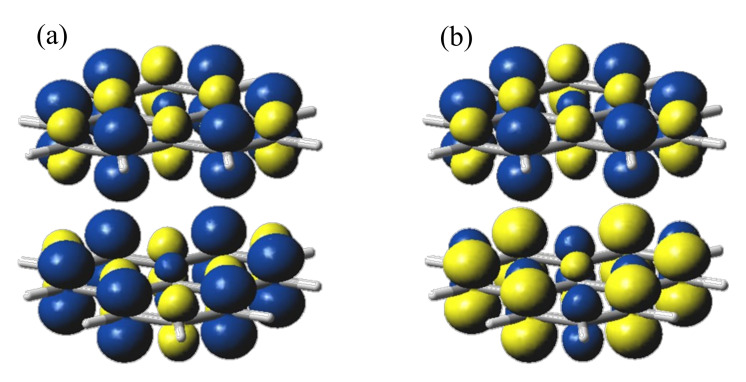
The spin density maps for (**a**) HS and (**b**) BS configurations of the phenalenyl dimer in staggered conformation (*D*_3d_ symmetry). The blue surfaces stand for α spin density and yellow for the β zones.

Figure 4 shows spin density maps for the staggered phenalenyl dimer in HS and BS configurations. One observes that, if labeled the monomers by 1 and 2 subscripts, the HS state corresponds to an α_1_α_2_ spin map, while the BS configuration corresponds to the α_1_β_2_ case, having the spin polarization reversed on the molecular unit represented in the bottom-right corner.

It is worth observing that for biradicals, the true ground LS energy can be computed in an indirect mode by the BS strategy. The obtainment of intermolecular coupling parameter *J_inter_* at each computed dimer geometry enables the emulation of the corresponding LS state, which is estimated as follows:

(10) $$E_{\mathrm{LS}}=E_{\mathrm{HS}}+2J_{inter}$$

Due to the fact that, in a spin-pairing paradigm, the realization of bonding (even in the weak long-range regime) corresponds to a negative coupling parameter, the obvious energy ordering is *E_LS_* < *E_BS_ < E_HS_*. The results are, obviously, dependent on the chosen functional and on the applied long-range correction, as is discussed later on.

### 2.3. The Fit of Potential Energy Profiles and Spin-Coupling Curves

A method to present synthetically the potential energy curves from the many performed tests relies on the fit parameters for a convened curve. The immediate choice can consist in Lennard–Jones, working with two parameters or Morse potentials [56,57], based on three variables, opting for the later one, in order to benefit from its greater flexibility:

(11) $$V_{M}\left(R\right)=D\cdot \left(e^{-2a\cdot \left(R-R_{0}\right)}-2e^{-a\cdot \left(R-R_{0}\right)}\right)$$

As is well known, the Morse potential is suited for describing curves with bonded minimums, acquiring the stabilization of −*D* at distance *R*_0_. During the tests, we met several situations where DFT computation does not render the expected minimum, and the potential curve showed only monotonous decay. For such a situation, a conventional method that can be used can be the so-called anti-Morse potential obtained by a sign swap in the below expression:

(12) $$V_{AM}\left(R\right)=D\cdot \left(e^{-2a\cdot \left(R-R_{0}\right)}+2e^{-a\cdot \left(R-R_{0}\right)}\right)$$

In order to account for any possibility in a single formula, a conventional mixture of Morse and anti-Morse curves was proposed [58]:

(13) $$V\left(R\right)=u\cdot V_{M}\left(R\right)+\left(1-u\right)\cdot V_{AM}\left(R\right)$$

Thus, with the cost of a supplementary parameter *u*, one may use the generalized Morse potential for bonded and non-bonded situations. However, the *u* mixing coefficient is not taken as a fitting parameter, since we found that it can be badly conditioned. In turn, we kept it fixed, *u* = 1 for regular Morse shape and *u* = 0 for anti-Morse, and used it merely as an indicator of the encountered situation. At the same time, the non-bonded curves, with a monotonous decay, are quite overparameterized in the presented anti-Morse form, since a simple exponential with two variables, an intra-exponential and a pre-exponential factor, can reasonably fit. To understand this, let us note that the dimension of saliently fitted parameter set must be comparable with the number of visually distinct features of a curve. For instance, in the Morse case, *R*_0_ and *D* correspond to the position and the depth of the minimum, while *a* tunes the steepness of the valley near minimum. Since, in the case of anti-Morse, *R*_0_ does not have the meaning of a minimum place, we chose to keep it fixed at a conventional value, *R*_0_ = 3.25 Å, when *u* = 0.

The Morse-type fit is applied on the data labelled with LS in the previous discussion. The exchange coupling is fitted with a simple exponential, as we employed in other instances [25,59]:

(14) $$J\left(R\right)=j\cdot e^{-p\cdot R}=J_{0}\cdot e^{-p\cdot \left(R-R_{0}\right)}$$

The formulation in the second equality, taking the *R*_0_ from the Morse fit, is convenient since the*J*_0_ pre-exponential factor measures the coupling at this distance. Otherwise, the first exponential form implies very large *j* factors with anon-physical meaning of an extrapolation at null distance.

For the sake of completeness, we present the fit for the quantity appearing in the denominator of Equation (1), which seems to obey the following pattern:

(15) $$\langle {\hat{S}}^{2}\rangle {}_{\mathrm{HS}}-\langle {\hat{S}}^{2}\rangle {}_{\mathrm{BS}}=\sigma_{1}+\frac{\sigma_{2}-\sigma_{1}}{2}\cdot \tan h\left(-s\cdot \left(R-\rho \right)\right)$$

σ_1_ and σ_2_ are parameters denoting minimal and maximal situations of Δ〈*S*^2^〉 expectation values. In all cases, σ_2_ is close to σ_2_ = 2;thus,it is kept fixed in the fit. In most cases σ_1_~1, but since there are certain malicious situations, it is taken as parameter of the fit. A proper BS regime has aΔ〈*S*^2^〉~1 signature, but as the distance is shortened and the dimer is enforced in the strongly bonded form, the trend to Δ〈*S*^2^〉 → 2 appears because 〈*S*^2^〉_BS_ turns to a null magnitude specific to closed shell situations. In principle, this situation is not desirable for BS calculation, but it is inescapable in the strongly bonded extreme. The *ρ* parameter has the formal meaning of interplanar separation situated at the midway between the ideal Δ〈*S*^2^〉 = 1 at long range and Δ〈*S*^2^〉 = 2 in the strong coupling regime. If *ρ* < *R*_0_, most of the points of the curve are close to ideal Δ〈*S*^2^〉 = 1 value, specific for singlet biradicals; otherwise, the system experiences gradual evolution toward a closed-shell electronic structure. The *s* parameter has no immediate intuitive meaning, providing a slope of the evolution between BS and closed shell regimes.

### 2.4. Genuine vs. Corrected Functionals. The B3LYP Tests

**Figure 5:**
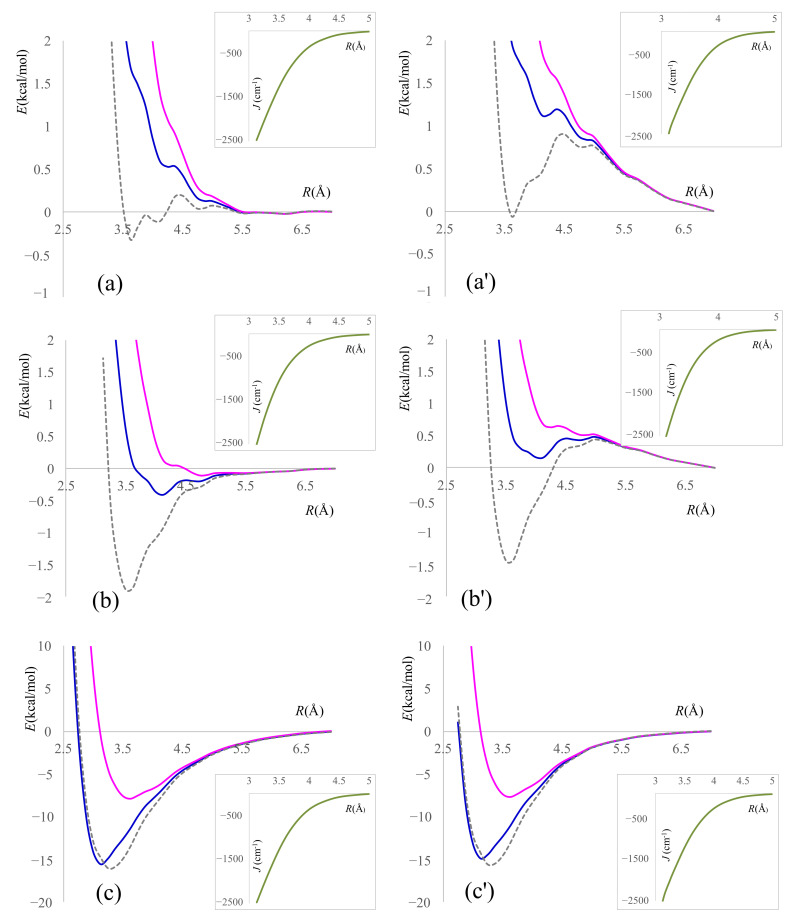
The unrestricted DFT energy curves for phenalenyl dimer in staggered conformation (*D*_3*d*_ symmetry), as a function of interplanar separation, *R*. Methods: (**a**) and (**a′**) by B3LYP, (**b**) and (**b′**) by CAM-B3LYP and (**c**) and (**c′**) by Grimme’s D3 correction to B3LYP;left side: 6-31+G* basis set; right side: def2TZVP basis set. The magenta (upper) curves correspond to triplet states (high spin—HS); the blue lines correspond to broken symmetry (BS) configuration. The dashed grey lines are singlet profiles (low spin—LS) emulated from triplet curve and the BS estimation of exchange coupling via Equations (1) and (10).

Before considering a benchmark with representative combinations of functionals [46,47,48,49,51,60,61,62,63,64,65,66] and long-range corrections [38,39,40,41], let us discuss the results derived from the most popular functional, B3LYP. Figure 5 shows potential energy curves for the association of phenalenyls in staggered conformation, comparing 6-31+G* [67] based calculations (the left side) with those from the def2TZVP basis set [68] (the right side), which is computed with three methods: genuine B3LYP;thedata amended with CAM (Coulomb Attenuated Method) [45]; and Grimme’s D3 increments [69]. The three different functionals worked with Pople-type basis are labeled a–c, while the related series with Ahlrichs orbitals correspond to a’–c’ series. The eclipsed conformation provides similar results that are not detailed here, since the staggered case is more important, according to its frequent occurrence in experimental instances [18,19].

A notable fact is that the variation of *J_inter_* exchange coupling with the separation of monomers (shown as inset in each panel from Figure 5) is almost the same in all cases, irrespective of the used long-range correction. Moreover, *J_inter_* curves seem almost insensitive to basis set, in which the checked two versions yielded closely superposable lines.

Then, what remains is assigning the details of the computed curves due to the effective spinless part of the underlying Hamiltonian. By contrast, this part is very dependent on the basis, noting severe differences in (a) vs. (a′) and (b) vs. (b′) panels, while (c) and (c′) are roughly comparable. The (a)–(a′) and (b)–(b′) couples show somewhat intriguing oscillatory behavior of all curves, probably due to the stepwise entering in long-range exchange regimes of different pairs from the symmetry-unique classes of the carbon atoms. In the (c)–(c′) pair, the Grimme correction, imposed outside of DFT calculation itself, probably supersedes the alleged effect. At the same time, if a higher positive abscissa range is allowed in (a), (a′) and (b) and (b′) representations, the oscillations become less visible. We checked carefully (tuning the numerical grid and levels of accuracy) that these are not due to numerical errors.

Focusing on the B3LYP case, without any long-range terms, one observes that both HS and BS curves show an exponential envelope, without bonded minimum, with both tested bases (Figure 5a,a′). However, if LS energy is used, as suggested in Equation (10), one gains a shallow minimum with the Pople-type basis and a strange pattern with the def2-type one. The last one has a deep-well appearance, while ported on an exponential decay profile, ending only with a very small area reaching the negative energies marking a bonded dimer. Working within CAM-B3LYP, the three curves from 6-31+G* calculation (Figure 5b) are obeying the intuitive manner of evolution: HS is non-bonded, as triplets in regular chemical bonds are expected, while BS shows a bonded minimum crossing the negative energy semi-plane, and the cohesion energy is larger for the LS curve. With the richer def2TZVP basis (Figure 5b′), the mutual placement and qualitative pattern of the curves are similar, but, in a closer detail, one observes that BS remains non-bonded, with positive energy, in spite of a local minimum. However, LS retrieves a stabilization that is semi-quantitatively similar to the precedent case from Figure 5b.

With the Grimme D3 corrections, the two bases behave similarly each to other (Figure 5c,c′), and all curves showed firmly bonded minima in the negative energy domain. The depth of LS minima is several times larger than in previous examples. Within the Grimme correction, BS and LS curves are comparable, a fact attributable to the large outer dispersion terms incorporated in the Grimme treatment in comparison to a smaller role of exchange itself. To this factor, one may assign the fact that the triplet state (HS) curve becomes a bonded minima in both Figure 5c,c′, which is a somewhat counterintuitive aspect.

However, the bonded triplet is not an artifactual result, since our previous investigations [25] with non-DFT methods proved the existence of triplet potential energy curve with the minimum as a physical truth. Partial conclusions from the above discussion suggest the Grimme-type alleviation as closer to the physical realism, curing drawbacks appearing in the pure B3LYP and incompletely resolved in CAM-B3LYP.

Ignoring the strong dependence of the stabilization energy on the setting of the functional, one may say that the minimum of LS curve is sketched in all cases at a reasonable value, around 3.5 Å. The experimental interplanar distance between phenalenyl rings, measured using the X-ray crystallography, varies between 3 and 3.5 Å, depending on the type of substituent groups. For example, if the three substituents are C_6_F_5_ groups, the separation is 3.503 Å (see reference [20]) and 3.111 Å with the phenyl substituted core [31].Even for the same chemical compound, the separation may vary due to different packing modes and crystal isomerism. For instance, for the system with three *tert*-butyl groups as substituents, the following different values are reported as 3.250 Å (in reference [70]) and 3.104 Å (in reference [71]).

### 2.5. Testing Selected Functionals and Long-Range Correction Recipes

In the following discussion we examine the phenalenyl dimer in different computational settings. The results are presented in synthetic form as parameters fitting the profiles of different intervening quantities, with functions defined in technical Section 3. Thus, the energy profiles of LS curves are considered with Morse curves when they show definite minima, while they are considered with so-called anti-Morse profiles when the show a non-bonded pattern (see Equation (12)). *J_inter_* exchange coupling is taken as exponential decay, vanishing with increasing intermolecular separation, *R* (see Equation (14)). The data on spin properties of the system are relevant, fitting the Δ〈S^2^〉 gap represented by the denominator of Equation (1) with a hyperbolic tangent function, i.e., a profile varying between upper and lower plateaus (see Equation (15)). With all these definitions one may retrieve, Equations (1) and (2) are the fitted forms of BS and HS curves. The spin-square expectation value of the monomer 〈*S*_0_^2^〉 resulting from each DFT setting is also relevant, and it is in the first numerical column of Table 1, Table 2 and Table 3. The tables compare two different bases with triple zeta and polarization: the medium-size Pople-type basis, 6-31+G* [67], and the larger set from Ahlrichs collection, def2TZVP [68].Ignoring, in first instance, the parametric details of Table 1, one may note that the magnitudes resulting from the 6-31+G* vs. def2TZVP calculations are quite comparable, and we can conclude from this that the Pople basis with diffuse and polarization primitives is sufficient in long-range problems.

In Table 1, we provide several uncorrected functionals belonging to different classes: the simplest levels of local density approximation (LDA), namely the Hartree–Fock–Slater (HFS) [60] having the classical Slater exchange functional. The following lines in Table 1 take two examples from the generalized gradient approximation class (GGA), namely BP86 (Becke–Perdew functional) [62,63] and BLYP (Becke–Lee–Yang–Parr) [46,47]. The hybrid methods are confined to the iconic example of B3LYP functional. The B3LYP data are, in principle, the same as those discussed in the previous section, while the fit included a larger energy window than represented in Figure 5. With this occasion, we note that the adopted Morse-type function for the LS is an approximation that may work reasonably in cases looking similar to Figure 5c,c′ snippets, as an acceptable trend in cases such as those from Figure 5a,b or quite rough in situations such as Figure 5a′,b′. In fact, in the last cases, one may better say that the DFT calculation is faulted, failing to render the expected Morse-like pattern. In the tables, *u* = 1 vs. *u* = 0 marks the use of Morse vs. anti-Morse profiles. In the anti-Morse case, the *R*_0_ parameter becomes superfluous, and it is conventionally fixed at 3.250 Å value, while *D* and *a* play other role than in the genuine bonded case. Inspecting the occurrence of *u* = 0, one may first note that it inflicts B3LYP calculation with def2TZVP basis (i.e., the case from above Figure 5a′), which shows no true stabilization of the dimer. Bad bonding conditions, enforcing anti-Morse regimes, are encountered with LC-uncorrected BLYP and B3LYP functionals.

By interpreting the parameters from Table 1, one may note the astonishingly good semi-quantitative play of low-level DFT methods, HFS and SVWN, yielding clean Morse-type LS curves, with large energy stabilization (*D*) at reasonable interplanar distances (*R*_0_). The exchange coupling strength at *R*_0_, namely the *J*_0_ pre-exponential factor is large, compared to the remaining examples of Table 1. This points to a firm long-range bonding obtained with uncorrected simplest functionals. There are no thermodynamic data affording an assessment of supramolecular formation energy, but one remarks *D* parameters as absolutely comparable with the well-rated case of B3LYP with Grimme D3 correction from the above discussion (if considering the visual depth of the minimum in Figure 5c,c′). The uncorrected B3LYP functional, shown at the bottom line of Table 1, performs badly, with a very shallow minimum (small *D* and large *R*_0_) under 6-31+G* basis and no minimum for def2TZVP, i.e., demanding an anti-Morse fit. The middle lines of Table 1 suggest that GGA functionals performed badly. BLYP yields non-bonded (anti-Morse) curves for both bases. BP86 shows weak bonding. In general, in all taken examples, large *D* parameters are correlated with smaller *R*_0_, both extrema being a signature for the relative strong bonding. Large *D* also seems conjugated with small *a*_0_ parameters, i.e., with larger vibrational energies, as a consequence of larger curvature at the minimum point. A glance at Table 1 shows that for LDA and GGA functional 〈*S*_0_^2^〉is relatively close to the 0.75 ideal value, while in the hybrid case it becomes slightly higher. Given the discussion from Section 2.1 pointing that Hartree–Fock results in large deviation 〈*S*_0_^2^〉, one may assign the B3LYP shift to the small portion of incorporated HF exchange.

The *J_0_* exchange parameters are roughly correlated with the magnitude of *D* in the case of Morse-type curves, in line with the idea that a part of the association comes from the spin-spin antiparallel coupling, such as in weak covalence, applied upon a background of van der Waals cohesion intrinsic to aromatic stacking in a spinless manner. The intra-exponential *p* parameters are comparable along the series. The last three columns are demonstrating that practically all systems are in a proper BS regime at the minimum point (where it exists) having *ρ* > *R*_0_ and *σ*~1. The *s* value is less intuitive, given here only for the sake of technical completeness.

Table 2 illustrates LC-type long-range corrections. In this strategy, the electron–electron potential, 1/*r_ee_*, is partitioned in short-range (SR) vs. long-range (LR) regions, equated by the respective first and second term in the following equation [39,40,41]:

(16) $$\frac{1}{r_{ee}}=\frac{1-\mathrm{erf}\left(\omega r_{ee}\right)}{r_{ee}}+\frac{\mathrm{erf}\left(\omega r_{ee}\right)}{r_{ee}}$$

where erf is the so-called standard error function, and *ω* is an adjustable parameter. The exchange energy is computed making the LR term object of a Hartree–Fock alike in terms of integration, while the DFT-type exchange is adapted to the SR operator. One should point that this formally simple dichotomy implies rather advanced modification of the technical scaffold, such as new formulas for exchange–correlation master formulas [42]. This makes LC calculation lengthier than its pure DFT form. One may say that LC turns all functionals into hybrid forms. Aside from LC being applied to LDA or GGA functionals, Table 2 exemplifies other specific adaptations of the range-separation idea, incorporated in the design of the functional, such as in CAM-B3LYP, M11 [48] and HSEH1PBE [64] cases.

First, one may note that in all range-separatedcalculationsthe 〈*S*_0_^2^〉 values are sensibly inflated, probably because of the enforced HF integration over the LR domain if considering the HF regime, which is the culprit for the alleged deviation in the squared spin averages, as discussed in Section 2.1.

A shocking effect of LC over LDA functionals is their mutation from good “adhesives” in phenalenyl dimers into “repellants,” where LC-HFS appear as anti-Morse with both bases, and the LC-SVWN is very loosely bonded. Conversely, GGA tests, which worked poorly in standalone modes, yield large *D* association energies and *R*_0_ values specific to stacked aromatic hydrocarbons. The particular LC-alike implementations from the last three lines of Table 2 yield medium or large association trends, suggesting that the effort to tailor new customized corrections may result in reasonable progress. *J*_0_ parameters show a lesser variance in the selection from Table 2, being grouped around a −5 kcal value, while, in Table 1, cases changed in a larger range. The LC recipe is expected to modify the exchange coupling, as long range-separation is explicitly addressed to this effect. The relatively good grouping of *J*_0_ and *p* parameters along this series can be interpreted by the fact that all LC calculations have a common amount, resulting from the Hartree–Fock integration taken over the LR term from Equation (16).

A glance at the last columns in Table 2 devoted to the fit of spin square data reveals that we encounter, almost systematically, situation *ρ* < *R*_0_, which is determined by certain perturbations in the ideal expectation values for the dimer, namely 〈*S*_HS_^2^〉 → 2 and 〈*S*_BS_^2^〉 → 1. This is induced with respect to the 〈*S*_0_^2^〉 deviation observed in monomer, and seems inescapable. However, in spite of such undesired irregularities, the differences forming the denominator of Equation (1) still are close to the idealized unitary value.

Let us proceed to the discussion of Table 3, collecting long-range corrections imposed by empirical addons at the top of the functional. The most popular choice is the Grimme-type treatment, the last version of this sort being consecrated with the D3 label. DFT energies are shifted with potentials that appear similar to generalizations of the Lennard–Jones prototype, driven by expansion over atom pairs (*AB*) and over inverse powers of their mutual distance, *r_AB_*, factored with certain damping functions, *f_d,n_* [49,50,51]:

(17) $$E_{\mathrm{disp}}^{\mathrm{two}-\mathrm{body}}=\underset{AB}{\sum }\underset{n=6,8,10,..}{\sum }\frac{s_{n}\left(C_{n}^{AB}\right)}{r_{AB}^{n}}f_{d,n}\left(r_{AB}\right)$$

*s_n_* scaling factors and *C_n_* dispersion coefficients are parameters supplementarily adjusted for the given atomic couple. This means that, aside from the empirical nature of the functional itself, we added further empirical ingredients. Although somewhat less elegant, this choice seems to be a practical method for inducing relatively stable long-range corrections, which are in principle independent from the selected functional.

The content of Table 3 shows parameters that are relatively comparable along the columns. For instance, *D* parameters are placed in the narrow range of about 15–22 kcal/mol, and equilibrium distances are in the 3.10–3.25 Å zone, describing firmly stabilized dimers. In this collection, all the systems have the *u* = 1 prefix, and they are well described by Morse curves. The exchange parameters at the equilibrium point, *J*_0_, are also relatively comparable and are placed in the 6–9 kcal/mol domain. In principle, in this class of methods, *J*_0_ and *p* parameters are not affected by Grimme correction terms, and they are then tuned only by the DFT core. Along the selected examples from Table 3, most encounter the *ρ* > *R*_0_ regularity, signaling that the system has a biradical nature along a good portion of the potential curve, with regular spin-square expectation values. The *ρ* < *R*_0_ swap observed at ωB97X-D functional can be assigned to the departure from spin-square encountered at the monomers, i.e., an overestimated 〈*S*_0_^2^〉 value. The other entries in Table 3 show slight deviations from the ideal 〈*S*_0_^2^〉 = 0.75 value.

## 3. Methods

### Computational Data

The impact of geometry variation as a function of basis and DFT definition is small with respect of dimerization energies and exchange coupling parameters. Consequently, in order to directly sense the role of the main computational frame, such as the dependence on the functional itself, we excluded secondary variances determined by relaxed potential energy shifts due to monomer part. The calculations were performed with the Gaussian 09 suite [72] comparatively by using Pople-type 6-31+G* [67] and def2TZVP Ahlrichs-type basis sets [68].GNU Octave [73] package was used for data analysis.

## 4. Conclusions

In addition to the electronic structure of the phenalenyl nucleus itself, a very interesting aspect is the long-range interaction between stacked phenalenyl cores, observed in all crystal structures of organic derivatives.

Since many empirical schemes of long-range corrections are calibrated on closed-shell electronic structures, the occurrence of spin–spin coupling in the biradical dimer brings into focus other effects than those met in standard benchmarks. Therefore, the actual study proposes new challenges with respect to long-range DFT. In this case, interplay occurs for exchange couplings and van der Waals forces.

BS configurations represent the DFT-type surrogate for singlet configurations in antiferromagnetically coupled biradicals. However, a low-spin (LS) energy, closer in meaning to the true singlet, can be emulated by combining the *J_inter_* parameter resulting from the BS-DFT procedure and HS energy: *E_LS_* = *E_HS_* − 2|*J_inter_*|. To the best of our knowledge, such aspects were not debated in the existing literature.

We systematically analyzed the dimeric system on a collection of selected functionals, which are grouped in three classes: (i) without long-range corrections; (ii) with a range-separated procedure (labeled generically LC) performed by conventional dichotomy of inter-electron potential; and (iii) by supplementary infusion of adjusting inter-atomic parameters, added at the top of a density functional calculation. The (i) branch of tests showed, that, although in general DFT has problems in describing supramolecular associations, an accidental cancelation of drawbacks may produce apparently good results in the case of very simple LDA functionals. The procedures inscribed in the (ii) class have the flavor of first-principles approaches to the problem. LC-type procedures may also provide certain alleviations, resulting in rather weak association energies in the cases of GGA and hybrid functionals, while destroying the accidental good performance of the simplest LDA methods. The (iii) series of the above classifications follows the empirical solution of adding corrective terms that resemble molecularly mechanic potentials. Adding more adjustable terms to the already empirical levels of DFT itself drives the treatment of inter-molecular problems to sloppy areas of multi-parametric dependencies. However, among the tested situations, these solutions seem to describe, in the best manner, the association of phenalenyl systems, validating this sort of pragmatic approach. Grimme-type increments are spinless; thus, these are addressed only in the dispersion part in our systems, and they do not affect exchange coupling components.

Looking across the collected data, one may observe a certain correlation between the *J*_0_ and *D* parameters: their absolute values are roughly parallel, although in a very scattered manner, with low correlation factors. In particular, a linear regularity is better kept for pure functionals (correlation *R*^2^~0.94) than for the corrected ones (*R*^2^~0.7). This note should be taken with reserve, since for a firmer conclusion must extend the studies. A possibly useful series of further numerical experiments would be a separate test of exchange or correlation parts of the functionals and related range amendments. A relationship between exchange and overall van der Waals cohesion seems intuitive, since, in stacked aromatic systems, both effects are driven by the same orbitals belonging to the π set of monomers. Then, a further and more elusive speculation can be suggested in using exchange couplings extracted from BS calculations (possibly on artificial radical objects) as sources for a new generation of long-range amendments.

In actual settings, starting from the premise that pure functionals from (i) series are intrinsically faulted, the corrections implied in the series labeled by (ii) remain rather unconclusive, while the procedures incorporated in (iii) are recommendable in the practical sense, although it is not the most glorious choice in terms of first principles.

We also tested the role of the basis, comparatively checking the 6-31+G* and def2TZVP sets. Although the latter one is, in principle, better rated than the former, the results of calculations in each couple are largely comparable throughout the stack of methods and for all outlined parameters.

## Figures and Tables

**Table 1:** Fit parameters * for phenalenyl dimer interaction in staggered configuration from processing the results of calculations with selected representative DFT methods, without long-range corrections.

Method	Basis Set	〈*S*_0_^2^〉	*u*	*GOF*	*D* kcal/mol	*R*_0_ (Å)	*a* (Å)^−1^	*J*_0_ kcal/mol	*p* (Å)^−1^	*ρ* (Å)	σ -	*s* (Å)^−1^
HFS	6-31+G*	0.764	1	0.9986	17.946	3.082	1.63895	11.152	1.79592	3.821	1.02399	3.41507
	def2tzvp	0.764	1	0.9968	17.759	3.051	1.73299	11.608	1.84319	3.929	1.02502	3.57648
SVWN	6-31+G*	0.756	1	0.9970	28.203	2.921	1.67267	15.594	1.7085	4.152	1.02147	3.32925
	def2tzvp	0.756	1	0.9952	28.063	2.906	1.70599	15.779	1.6113	4.142	1.02151	3.44984
BP86	6-31+G*	0.767	1	0.9871	2.510	3.329	2.21563	6.807	1.98562	3.712	1.01870	3.34216
	def2tzvp	0.767	1	0.9636	1.439	3.362	2.44780	6.657	2.08065	3.706	1.02009	3.61832
BLYP	6-31+G*	0.765	0	0.9873	0.773	3.250	3.59274	7.549	2.10370	3.447	1.01908	3.61409
	def2tzvp	0.765	0	0.9689	1.042	3.250	3.16227	7.502	2.15263	3.446	1.01832	3.63673
B3LYP	6-31+G*	0.799	1	0.9933	0.318	3.837	2.41695	2.598	2.24661	3.447	1.01907	3.61409
	def2tzvp	0.798	0	0.9587	1.115	3.250	3.35913	5.729	2.13559	3.446	1.01832	3.63673

* The first numerical column prints the 〈*S*_0_^2^〉 obtained value for the phenalenyl monomer as representing the overall quality of the calculation. The following columns refer to the Morse curve(see Equations (11)–(13)): *u* = 1 marks the use of regular Morse curve (with minimum) and *u* = 0 stands for antibonding form (no minimum). At *u* = 0, the *R*_0_ value is conventionally fixed at 3.25 Å; *GOF*—goodness of fit, the correlation coefficient for the linear representation of computed vs. fitted potential; *D*—the absolute value of energy stabilization; *R*_0_—the equilibrium interplanar distance; *a* is the coefficient inside the exponential Morse term. *J*_0_ and *p* are parameters of the exponential fit of intercenter spin-coupling strength *J* (see Equation (14)). *ρ*, *σ* and *s* are fitting the Δ〈*S*^2^〉 gap (the denominator of Equation (1)) with a shifted and scaled hyperbolic tangent pattern (see Equation (15)). The acronyms in first column are the corresponding Gaussian input keywords.

**Table 2:** Bonding and spin Hamiltonian parameters from handling data from range-corrected functionals. The meaning of the quantities is the same as in Table 1.

Method	Basis Set	〈*S*_0_^2^〉	*u*	GOF	*D* kcal/mol	*R*_0_ (Å)	*a* (Å)^−1^	*J*_0_ kcal/mol	*p* (Å)^−1^	*ρ* (Å)	*σ* -	*s* (Å)^−1^
LC-HFS	6-31+G*	1.144	0	0.9983	3.480	3.250	2.64500	4.481	2.90744	3.821	1.02399	3.41507
	def2tzvp	1.141	0	0.9906	2.527	3.250	1.89001	4.543	2.93621	3.929	1.02502	3.57648
LC-SVWN	6-31+G*	0,979	1	0.9981	0.658	3.906	1.72638	0.863	2.52575	2.876	1.00203	1.97545
	def2tzvp	0,977	1	0.9849	0.265	3.848	2.33551	0.919	2.68017	2.841	1.00091	1.83531
LC-BP86	6-31+G*	1.030	1	0.9973	12.108	3.105	2.13143	6.931	2.16669	3.026	1.01035	3.21305
	def2tzvp	1.028	1	0.9927	10.304	3.116	2.10700	6.484	2.24239	2.937	1.00807	2.66578
LC-BLYP	6-31+G*	1.010	1	0.9920	5.423	3.355	1.72612	3.679	2.5073	2.784	0.99893	1.57951
	def2tzvp	1.006	1	0.9928	5.253	3.324	2.00463	3.489	2.69383	2.897	1.00361	2.10122
CAM-B3LYP	6-31+G*	0.868	1	0.9780	4.450	3.391	2.24565	7.174	2.24928	3.144	1.00777	2.58372
	def2tzvp	0.867	1	0.9796	1.201	3.499	2.61995	3.073	2.4510	3.106	1.00352	2.14649
HSEH1PBE	6-31+G*	0.823	1	0.9893	5.114	3.329	1.98489	5.122	2.03229	3.260	1.01873	3.73800
	def2tzvp	0.823	1	0.9949	4.181	3.301	2.46109	5.402	2.21272	3.258	1.01849	3.80583
M11	6-31+G*	0.879	1	0.9931	16.903	3.145	1.82830	6.020	2.05099	3.080	1.01722	4.35074
	def2tzvp	0.872	1	0.9956	17.319	3.075	1.93448	7.728	2.03871	2.971	1.01641	4.18583

**Table 3:** Bonding and spin Hamiltonian parameters from handling data from DFT calculations incremented with built-in dispersion addons. The meaning of the quantities is the same as in Table 1.

Method	Basis Set	〈*S*_0_^2^〉	*u*	GOF	*D* kcal/mol	*R*_0_ (Å)	*a* (Å)^−1^	*J*_0_ kcal/mol	*p* (Å)^−1^	*ρ* (Å)	*σ* -	*s* (Å)^−1^
B3LYP GD3	6-31+G*	0.799	1	0.9988	15.982	3.256	1.41408	5.926	2.14210	3.447	1.01908	3.61409
	def2tzvp	0.798	1	0.9977	15.496	3.247	1.48886	5.798	2.19535	3.446	1.01832	3.63673
B97D	6-31+G*	0.773	1	0.9938	21.785	3.094	1.56362	8.979	2.00851	3.729	1.02388	3.57989
	def2tzvp	0.774	1	0.9984	21.118	3.111	1.54651	9.167	1.93595	3.593	1.02162	3.67610
B97D3	6-31+G*	0.773	1	0.9990	23.942	3.101	1.43868	9.323	1.86482	3.249	1.01954	3.30823
	def2tzvp	0.774	1	0.9995	22.961	3.105	1.46734	9.167	1.93595	3.594	1.01819	3.34281
wB97XD	6-31+G*	0.850	1	0.9990	19.668	3.197	1.59813	6.366	2.0404	3.043	1.01459	3.56559
	def2tzvp	0.851	1	0.9993	18.308	3.199	1.64489	6.243	2.07563	3.041	1.01441	3.60865
APFD	6-31+G*	0.818	1	0.9971	23.665	3.135	1.50090	7.676	2.03858	3.249	1.02002	4.04944
	def2tzvp	0.817	1	0.9976	22.455	3.135	1.55166	7.592	2.06477	3.249	1.01938	4.06371

## Data Availability

Details are available from corresponding authors.
